# Protective effect of *Cassia glauca* Linn. on the serum glucose and hepatic enzymes level in streptozotocin induced NIDDM in rats

**DOI:** 10.4103/0253-7613.48887

**Published:** 2009-02

**Authors:** Mamta Farswan, Papiya Mitra Mazumder, V. Percha

**Affiliations:** 1 Department of Pharmaceutical Sciences, SBS (PG) Institute of Biomedical Sciences, Balawala, Dehradun, India; 2 Birla Institute of Technology, Mesra, Ranchi, Jharkhand, India

**Keywords:** β-sitosterol, blood glucose, *Cassia glauca*, hepatoprotective

## Abstract

**Objective:**

The objective of the present study was to investigate the hypoglycemic and hepatoprotective effect of *Cassia glauca* leaf extracts on normal and non insulin dependent diabetes mellitus (NIDDM) in rats. The study was further carried out to investigate the effect of different fractions of the active extract of *Cassia glauca*, on normal and NIDDM rats, and the effect of active fraction on the blood glucose and hepatic enzymes level.

**Methods:**

Diabetes was induced by streptozotocin (STZ) at a dose of 90mg/kg, i.p. in neonates. Different extracts of *cassia glauca* (100mg/kg, p.o.) were administered to the diabetic rats. Acetone extract was found to lower the serum glucose level significantly in diabetic rats. Further, the acetone extract was subjected to column chromatography and four fractions were obtained on the basis of TLC. All the four fractions (100mg/kg, p.o.) were administered to the diabetic rats. Fraction 1 (F1) caused the maximum reduction in the blood glucose level. The results of the test were compared with the standard antidiabetic drug glibenclamide (5mg/kg, p.o.).

**Results:**

Fraction 1 of acetone extract caused a significant reduction in the levels of hepatic enzyme Aspartate transaminase (AST), alanine transaminase (ALT), creatine kinase (CK), and lactate dehydrogenase (LDH) in STZ-induced diabetic rats.

**Conclusion:**

Improvement in the blood sugar level and normalization of liver functions by *Cassia glauca* indicates that the plant has hepatoprotective potential, along with antidiabetic activity, and it provides a scientific rationale for the use of *Cassia glauca* as an antidiabetic agent.

## Introduction

There is still an unmet need for medicinal plants and phytopharmaceuticals with scientifically proven antidiabetic activity. Diabetes mellitus is characterized by a progressive decline in insulin action (insulin resistance, followed by the inability of β cells to compensate for insulin resistance.[1] The β cells normally compensate insulin resistance by secreting more amount of insulin to maintain the glucose homeostasis. In non insulin dependent diabetes mellitus (NIDDM) this β cell function gets impaired leading to deterioration in glucose homeostasis and subsequent development of impaired glucose tolerance and frank diabetes.[2,3] Hyperglycemia in the diabetics is associated with alteration of glucose and lipid metabolism and modification in liver enzymes level[4].

Liver is an important insulin dependent tissue which plays a pivotal role in glucose and lipid metabolism and is severely affected during diabetes.[5] In diabetes the levels of hepatic enzymes increases. The level of ALT increases due to hepatocellular damage and is always accompanied by an increase in AST activity. Moreover the ALT and AST activity has been used as an indicator of liver function.[6]

The prevalence of diabetes is approximately 3% of the world population and is about 5% in the United States.[7] Approximately 10% of patients have type 1 diabetes mellitus (DM) and the remainder have type 2 Diabetes Mellitus.

Insulin and oral hypoglycemics are the most widely used drugs for diabetes but they also have various side effects like hypoglycemia, weight gain (sulfonylurea), lactic acidosis with biguinides and all of these drugs may cause liver and renal damage.[8]

From the beginning of last century, evidence of lipid lowering properties of medicinal plants has accumulated.[9] In recent years many traditionally used medicinal plants have been tested for their antidiabetic potential in experimental animals.[10] Ethnobotanical information indicates that more than 800 plants are used as traditional remedies for the treatment of diabetes.[11]

*Cassia glauca* (Family – Caesalpiniaceae) is about 10 ft high, evergreen shrub. Aerial parts of the plant are used as central nervous system depressant and as a diuretic.[12] Seeds of the plant are known to possess purgative and antimalarial properties. The leaves of the plant are pounded in sugar and milk and used to cure blennorrhagia. Bark and leaves are used in diabetes and gonorrhea in the folk lore medicine. This plant is also a good pollution tolerant and reduces chemical pollutants from the atmosphere.[13] Phytochemical investigation of the plant showed the presence of γ- sitosteroline, fatty acids, anthraquinones, tannins and alkaloids. The leaves contain β -sitosterol and lupeol. Different species of Cassia like *Cassia alata*,[14] *Cassia auriculata*[15] have also been studied well for their antidiabetic potential. Therefore the objectives of the present study were to evaluate the hypoglycaemic and hepatoprotective activity of active fraction of *Cassia glauca* leaf extracts on normal and NIDDM rats. The results obtained with *Cassia glauca* were compared with glibenclamide, a commonly used hypoglycaemic agent.

## Materials and Methods

*Cassia glauca* leaves were collected from the forest of Dehradun. The plant was identified, authenticated and the voucher specimen (A-30) has been kept at the herbarium of Sardar Bhagwan Singh (PG) Institute of Biomedical Sciences Dehradun. Streptozotocin was purchased from Calbiochem, Germany. Glibenclamide was obtained as gift samples from Ranbaxy Research Laboratories. Analytical grade chemicals like various organic solvents (petroleum ether, chloroform, acetone, and methanol) from E. Merck India Ltd and Ranbaxy were used for the extraction and phytochemical study of the constituents.

### Preparation of Plant extracts

Fresh plant leaves were shade dried at room temperature, ground into fine powder, and then extracted (amount 450 gm) by solvents, petroleum ether, chloroform, acetone and methanol in increasing order of polarity of the solvents, for 24 h with each solvent by soxhlet extraction at a temperature of 60°C. After extraction the solvents were removed from the extracts (petroleum ether extract, chloroform extract, acetone extract and methanol extract of *Cassia glauca* leaf) under reduced pressure using a rotary evaporator. The extracts were collected and preserved in a desiccator until used for further studies.

### Phytochemical study

All the extracts of the leaf of *cassia glauca* were screened for their phytoconstituents in order to see the chemical nature of the compounds present in the extracts. A portion of residue from each extract was subjected to phytochemical analysis in order to see the presence of sterols, alkaloids, carbohydrates, tannins, phenols etc in the extracts.[16,17]

### Fractionation of the acetone extract of Cassia glauca leaf

All the extracts of *Cassia glauca* leaf were screened for antidiabetic activity in diabetic rats. Acetone extract was found to show maximum reduction in blood sugar level, therefore attempts were made to isolate the active principle from the active acetone extract. The acetone extract (10 gm) was thus subjected to column chromatography using silica gel mesh (200-400) size and using CHCl_3_: MeOH in the increasing order of polarity as solvent. Based on thin layer chromatography (SiO_2_ and CHCl_3_) four fractions (F1, F2, F3, F4) were obtained from the active extract. These fractions were screened for antidiabetic activity. Fraction 1 showed maximum antidiabetic activity.

### Determination of blood glucose and lipid profile in diabetic rats

*Animals:* Wistar albino rats of either sex were randomly bred in the Institutional animal house. The animals were housed in standard polypropylene cages and maintained under controlled room temperature(22 ± 2°C) and humidity (55+5%) with 12:12 hour light and dark cycle. All the animals were provided with commercially available rat normal pellet diet and water ad libitum. The guidelines of committee for the purpose of control and supervision of experiments on animals (CPCSEA) Govt. of India were followed and prior approval was taken from the institutional animal ethics committee for conducting the animal experimental studies.

### Acute toxicity studies

Acute toxicity studies were carried out on Swiss albino mice by the method of Ghosh.[18] Active acetone extract at a dose of 100, 300, 500, 1000 and 3000 mg/kg was administered to separate groups of mice each having 6 animals. After administration of extracts the animals were observed for first 3 hour for any toxic symptoms followed by observation at regular intervals for 24 hour up to 7 days. At the end of study the animals were also observed for general toxic signs, morphological behaviour and mortality.[19]

### Induction of Diabetes

The method of Portha et. al was followed for the induction of diabetes. To induce type II diabetes, five day old Wistar neonates were injected intraperitonially with 90 mg/kg streptozotocin in 0.1M citrate buffer pH 4.5.[20] The control group received equivalent amount of citrate buffer. The pups were allowed to be with their respective mothers and weaned at 4 weeks of age. Eight weeks after injection of streptozotocin, the rats were checked for fasting blood sugar (FBS) level.[21] The animals showing FBS more than 150mg/dl were considered as diabetic and included for the study.

### Treatment protocol

The diabetic animals were divided into five groups each having six animals. All the extracts were given at a dose of 100mg/kg, p.o., for a period of 15 days.

Group I: Normal animals received tween 80 in a dose of 1% suspension in distilled water.

Group II: Diabetic animals received tween 80 in a dose of 1% suspension in distilled water.

Group III: Diabetic animals received glibenclamide in a dose of 5 mg/kg, p.o.

Group IV: Diabetic animals received Petroleum ether extract in a dose of 100 mg/kg, p.o.

Group V: Diabetic animals received chloroform extract, in a dose of 100 mg/kg, p.o Group VI: Diabetic animals received acetone extract, in a dose of 100 mg/kg, p.o

Group VII: Diabetic animals received methanol extract. in a dose of 100 mg/kg, p.o.

At the end of experimental period the animals were fasted overnight and blood was taken from the retro orbital plexus under mild ether anesthesia, serum was separated out and blood sugar level was evaluated by the method of glucose oxidase- peroxides method using Span diagnostic kits.

### Preliminary pharmacological screening of the fractions and effect of active fraction on hepatic enzymes level in diabetic rats

Diabetic animals were divided into five groups of five animals in each.

Group I: Normal animals received tween 80 in a dose of 1% suspension in distilled water.

Group II: Diabetic animals received tween 80 in a dose of 1% suspension in distilled water.

Group III: Diabetic animals received glibenclamide (5mg/kg),

Group IV– VII: diabetic animals received Fraction1 (F1) to fraction 4 (F4) respectively in a dose of 100mg/kg, p.o, for a period of 15 days.

At the end of experimental period animal were fasted overnight and blood was taken from the retro orbital plexus under mild ether anesthesia, it was centrifuged and serum was separated out. Effect of each fraction on FBS level was evaluated. Fraction 1 caused maximum reduction in FBS in diabetic rats. Serum from the group receiving fraction 1 was taken to study the effect of fraction 1 on the lipid profile. AST, ALT, CK and LDH were determined by UV kinetic method^22^ using span diagnostic kits. Results of the test were compared with that of the standard antidiabetic drug glibenclamide.

### Statistical analysis

The results were expressed as Mean ± SEM. The unpaired t-test was used for analyzing the data between two groups. Statistical analysis of data was initially performed by using analysis of variance (ANOVA), when the overall ANOVA was significant, tukeys test for significance was applied to study the difference among the groups.

## Results

### Phytochemical study

Petroleum ether extract of the leaf showed the presence of sterols. Chloroform extract showed the presence of carbohydrates & alkaloids. Acetone extract showed the presence of carbohydrates & alkaloids and tannins. Methanol showed the presence of tannins, and alkaloids. Water extract showed the presence of carbohydrates and glycosides.

### Acute toxicity studies

Acute toxicity studies revealed that *Cassia glauca* extracts were practically non toxic when administered orally to mice. The LD50 value was 3gm/kg body weight.

Effect of *Cassia glauca* leaf extracts and Fractions of active extract on fasting blood sugar and hepatic enzymes level in diabetic rats.

Table 1 illustrates the effect of different extracts of *Cassia glauca* on serum glucose level in the diabetic rats. Results showed that all the extracts caused reduction in blood glucose level but maximum reduction was found in acetone extract. Acetone extract showed 52% reduction as compared to glibenclamide (67%) reduction in fasting blood sugar. Acetone extract of the leaf caused significant (p<0.01) decrease in the fasting blood glucose. Table 2 illustrates the effect of different fractions of acetone extract of *Cassia glauca* on serum glucose level in the diabetic rats. Results showed that all the fractions of acetone extract of the leaf caused significant decrease in the fasting blood glucose. However F1 exhibited maximum reduction in fasting blood sugar (59%) as compared to glibenclamide which caused 67% reduction in FBS.

**Table 1:** The effect of *Cassia glauca* leaf extracts on fasting blood sugar of diabetic rats (n = 6)

*Groups reduction*	*Blood sugar*		*%*
		
	*before treatment*	*after treatment*	*in blood sugar*
Control			
(diabetic rats)	241±1.3	235±0.8	-
Normal			
(tween 80 1ml/kg, p.o)	90±0.8	96±1.1	-
Diabetic + Standard drug			
(5 mg /kg, p.o.)	260±1.1	90±1.3***	67
Diabetic + Pt. ether			
(100mg/kg, p.o)	229+1.3	130±1.1*	35
Diabetic + Chloroform			
(100mg/kg, p.o.)	236±1.7	134±1.3*	43
Diabetic + Acetone			
(100mg/kg, p.o.)	224±2.6	107±1.4**	52
Diabetic + Methanol			
(100mg/kg, p.o.)	220±3.8	129±1.5*	41

Results were expressed as Mean ± SEM. Results of the test and standard groups were compared with the control group.

* *P*<0.05

** *P*<0.01

*** *P*<0.001

**Table 2:** The effect of fractions of active extract (acetone) of *Cassia glauca* on fasting blood sugar of diabetic rats (*n* = 6).

*Groups*	*Blood sugar before treatment*	*Blood sugar after treatment*	*% reduction in blood sugar*
Control	241 ± 1.3	235 ± 0.8	-
(diabetic rats)			
Normal	90 ± 0.8	96 ± 1.1	-
(tween 80 1ml/kg, p.o)			
Diabetic + Standard drug	260 ± 1.1	90 ± 1.3***	67
(5 mg/kg, p.o.)			
Diabetic + F1	235 ± 0.8	96 ± 0.8**	59.2
(100mg/kg, p.o)			
Diabetic + F2	200 ± 1.1	115 ± 2.0*	42
(100mg/kg, p.o.)			
Diabetic + F3	210 ± 2	119 ± 1.0*	44
(100mg/kg, p.o.)			43
Diabetic + F4	210 ± 2	116±0.7*	
(100mg/kg, p.o.)			

Fraction 1=F1, Fraction 1=F2, Fraction 1=F3, Fraction 1=F4

Results were expressed as Mean ± SEM.

Results of the test and standard groups were compared with the control group.

* *P*<0.05

** *P*<0.01

*** *P*<0.001

Table 3 illustrates the effect of F1 and glibenclamide on AST, ALT, Creatine kinase and lactate dehydrogenase level. Fraction 1 of the acetone extract caused significant (p<0.01) improvement in AST, ALT, CK and LDH levels in the diabetic rats after treatment.

**Table 3:** The effect of fraction 1 of acetone extract of *Cassia glauca* on lipid profile in diabetic rat (*n* = 6).

*Groups*	*AST (units/ml of serum)*	*ALT (units/ml of serum)*	*Creatine kinase (IU/L)*	*Lactate Dehydrogenase (IU/L)*
Normal				
(1ml/kg, p.o.)	40 ± 0.8	36 ± 0.5	196 ± 0.8	207 ± 0.8
Control				
(diabetic rat)	86 ± 1.4	87 ± 0.4	913.3 ± 9	754 ± 4.3
Diabetic +Std.				
(5 mg/kg, p.o.)	42 ± 0.8 **	37.9 ± 0.4 ***	212 ± 0.8**	223 ±0.7**
Diabetic + F1				
(100mg/kg, p.o.)	59 ± 0.36**	60 ± 0.1 *	271 ± 3*	248 ± 1.1**

Results were expressed as Mean ± SEM.

Results of the test and standard groups were compared with the control group.

* *P*<0.05

** *P*<0.01

*** *P*<0.001

ALT: Alanine transaminase, AST: Aspartate transaminase, CK: Creatine kinase, LDH: Lactate dehydrogenase

## Discussion

In the recent years various plant extracts have been claimed to be useful for the treatment of diabetes.[23] Administration of STZ caused rapid destruction of β cells which lead to a reduction of insulin release which slowly progress to impaired glucose stimulated insulin release and insulin resistance which are the marked feature of type II diabetes. The hypoglycemic effect of plant extracts is generally dependent upon the degree of β cell destruction. In diabetes, the increase in blood glucose level is usually accompanied by an increase in the activity of transaminases. More over the ALT, AST and LDH activities has been used as an indicator of liver function. Acetone extract caused significant reduction in the serum blood glucose level as compared to glibenclamide. Acetone extract was subjected to column chromatography in order to get fractions containing maximum hypoglycemic activity. Fraction 1 caused reduction in blood sugar which is more than the acetone extract. Fraction 1 also caused 31% reduction in the level of AST and 32% reduction in the level of ALT where as glibenclamide caused 23% reduction in the level of CK and 20% reduction in the level of LDH. The improvements in the levels of liver enzymes in diabetic animals could be beneficial in preventing diabetic complications, as well as improving lipid and protein metabolism in diabetic liver. The reversal of AST and ALT activity in *Cassia glauca* treated diabetic rats towards near normalcy is evidence of the prevention of cellular and tissue damage under diabetic conditions.

Phytochemical investigation of *Cassia glauca* leaf showed the presence of γ-sitosteroline, fatty acids, anthraquinones, tannin and alkaloids. On the chemical basis it may be concluded that β - sitosterol and tannins in the leaf are responsible for the antidiabetic activity of *Cassia glauca* as it is known that tannins and sterols containing drugs possess antidiabetic activity.[24]

The present investigation revealed that acetone extract and fraction 1 of the acetone extract of *Cassia glauca* leaf has shown significant antidiabetic activity.

Improvement in the liver enzymes level by *Cassia glauca* indiates that the plant has hepatoprotective potential along with antidiabetic activity. The active constituent in the fraction responsible for antidiabetic and hepatoprotective activity is not known at present and work is on process to isolate it.
